# A tissue multiplexing approach to maximize spatial omics output from pathology specimens

**DOI:** 10.3389/fmed.2026.1749401

**Published:** 2026-04-09

**Authors:** Yung-Ching Kao, Katie J. Lee, Chenhao Zhou, Andrew Causer, Harald Oey, Kiarash Khosrotehrani, Blake O’Brien, Angus Collins, Quan Nguyen, H. Peter Soyer, Mitchell S. Stark

**Affiliations:** 1 Frazer Institute, The University of Queensland, Dermatology Research Centre, Brisbane, QLD, Australia; 2 Institute for Molecular Bioscience, the University of Queensland, Saint Lucia, QLD, Australia; 3 Department of Dermatology, Princess Alexandra Hospital, Brisbane, QLD, Australia; 4 Sullivan Nicolaides Pathology, Brisbane, QLD, Australia; 5 QIMR Berghofer Medical Research Institute, Herston, QLD, Australia

**Keywords:** multiplex * diagnostics, skin, spatial transcriptomics, tissue, transciptomics

## Abstract

Spatial profiling technologies are revolutionising traditional research by enabling whole transcriptome and high-plex protein *in situ* analysis at scale. Profiling tissue microarrays permits multiple specimens to be analysed at once, but depending upon your research question, these are not widely applicable. Since diagnostic specimens retrieved from pathology laboratories cannot be altered (aside from sectioning) for ethico-legal reasons, this method introduces an approach in which multiple patient specimens, sectioned from individual tissue blocks, are combined to enable measurement of several tissues within a single capture array. This strategy provides a cost-effective means to increase sample size, permitting statistically robust data outputs, and fast-tracks clinical implementation. Importantly, our tissue multiplexing method is compatible with the standard protocols of multiple spatial platforms, including Visium CytAssist (and HD), Xenium *in situ*, GeoMx, CosMx, PhenoCycler-Fusion, and MERSCOPE. We have used skin biopsies in this study as this is our primary area of research, but this method can be extended to any tissue of interest.

## Introduction

Cutting-edge spatial transcriptomic (ST) and protein profiling is a technology that enables spatially resolved whole transcriptome detection and high-plex proteins (depends on antibody availability and panel design) across fresh frozen and formalin-fixed paraffin-embedded (FFPE) tissue sections (1, 2). This technology reveals positional context of cell populations and their interactions, which is essential for improving our understanding of disease progression and treatment efficacy (3). Common spatial platforms include Visium V1, GeoMx, CosMx, Visium HD, Xenium *in situ*, PhenoCycler-Fusion (4) and MERSCOPE that enable the understanding of molecular insights underlying pathology sections. Regardless of whether the technology utilizes probe-based or imaging-based method, common limitations to these platforms include a relatively small capture area for tissue placement, long processing time of individual samples, combined with the high financial costs for reagents and sequencing.

In most cases, if diagnostic tissue blocks can be retrieved from pathology for research purposes, these cannot be used to generate a tissue microarray or removed in its entirety from the original block due to medico-legal and ethical constraints. Moreover, each paraffin block often contains multiple tissues generated from the same biopsy, and upon hematoxylin and eosin (H&E) review of sections, not all tissues contain the area of interest. The power of the present method is the ability to include only the areas of interest in the small capture area, and this method is applicable to various platforms as noted. This is particularly useful when only tissue sections are available from pathology, or if a smaller region of a large tissue biopsy is the focal point. Herein, we describe our experience processing skin biopsies using the Visium CytAssist (10x Genomics) platform as an example. We describe a modified tissue preparation workflow that maximizes the tissue section coverage of the capture area (6.5 × 6.5 mm or 11 × 11 mm window), as a cost-effective strategy to increase sample size.

**Figure 1:**
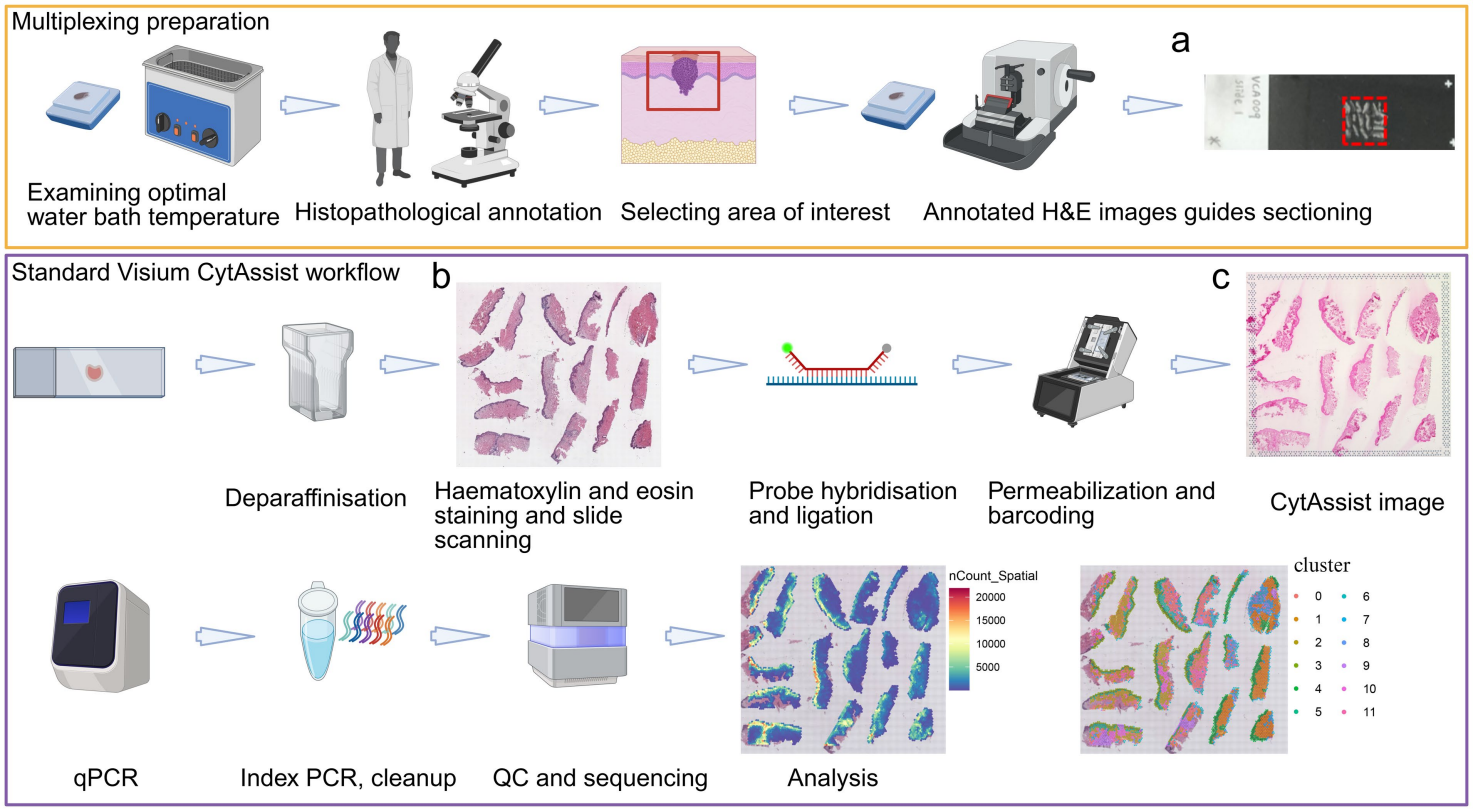
Illustration of the multiplexing of 17 skin tissues in one 11 mm × 11 mm Visium capture area. Multiplexing tissues for Visium CytAssist workflow requires optimizing the water bath temperature for each sample, selecting the area of interest, and isolating the area of interest by performing dissection on a 4 μm section with a scalpel blade (on a microtome stage) or forceps (in a water bath). After incubation in a water bath, the isolated area of interest is placed as closely as possible within a pre-drawn 11 × 11 mm designated square (outlined in red) on a positively charged tissue slide **(a)**. This step is repeated for each sample until the pre-drawn 11 × 11 mm area is covered entirely. Compatible tissue slides and allowable area for CytAssist assay are detailed in the sample preparation protocol CG000520 (10× Genomics). Once the tissue slides containing the FFPE tissue sections are dried overnight in a desiccator, the tissues are stained with hematoxylin and eosin (H&E) and scanned **(b)** following protocol CG000520 (10× Genomics). The H&E-stained slide is then subjected to destaining, probe hybridization, probe ligation as per protocol CG000495 (10× Genomics). Once the processed tissue slide is ready for CytAssist-enabled transfer, the slide is stained again with eosin to mark the tissue regions to match the Visium CytAssist capture area. As part of the CytAssist-enabled transfer, the gene expression probes are released from the tissues and captured by the spatially barcoded oligonucleotides on the Visium slide. An image is taken using the CytAssist instrument and superimposed onto the capture area which permits later H&E image alignment relative to the spatially resolved spots **(c)**. After the CytAssist transfer, the Visium slide is subjected to the standard workflow as per protocol CG000495 (10× Genomics). To generate the web summary output from a multiplexed capture area, manual selection of Visium spots covered by each tissue sample on Loupe Browser (V7 or higher; 10× Genomics) is required. This will allow the alignment of the spatially resolved spots with H&E/CytAssist image. Our multiplexed array demonstrated higher gene counts in the epidermis/epidermal-dermal junction across all skin samples. Clustering analysis confirms that the same cluster is assigned to the epidermis for all 17 samples. For downstream analyses, each tissue within the capture area, as visualized on the Loupe Browser, is saved as individual file to enable independent analysis. Created with BioRender.com.

Skin biopsies (including lesions suspicious for melanoma or keratinocyte cancer) excised for diagnostic purposes vary in size (~2–10 mm^3^ to multiple cm^3^), and previous ST has highlighted that most genes are detected in the epidermis and dermo-epidermal junction (5), and are usually sparse in the dermis or subcutaneous tissue (6). As such, if only one tissue section per slide is used, this may only cover ~10% of the capture area which is an inefficient use of these powerful tools. This can be addressed by combining many tissue specimens and regions of interest, cut from multiple tissue sections, into a single tissue slide (Figure 1a). Relative to the Visium CytAssist procedure, isolated regions of interest are placed within the same sized capture area on a standard positively-charged glass slide, to maximize transfer of multiple tissues into one capture area of the corresponding Visium slide. Depending on the profiling platform, the tissue regions of interest may be placed directly onto the platform’s imaging slide.

## Materials and methods

We provide some key recommendations to achieve tissue multiplexing prior to commencement of the standard workflow in the 10x Genomics Demonstrated Protocol #CG000520. These methods were developed to enable spatial profiling in a more cost and time-efficient manner and has been applied for both spatial transcriptomic and multiplex protein profiling.

### Step1: determining optimal water bath temperature

Prior to sectioning tissues for spatial profiling, it is important to record the optimal water bath temperature by observing if the tissues flatten completely when floated in a water bath, without section disintegration. Tissue disintegration may result from the age of tissue blocks, variable fixation times, tissue of origin, or the presence of abundant adipose cells. The optimal water bath temperature must be reduced if tissues disintegrate once floated. In our cohort, FFPE tissue block ages ranged from 1 to 20 + years, with water bath temperatures ranging from 30 to 40 °C.

We used a freshly cut 4 μm section, as opposed to the recommended 5 μm thickness for our skin sections, since the thicker section resulted in a high level of tissue detachment during probe hybridization and eosin wash (part of standard workflow Protocol #CG000520). Once the optimal water bath temperature is reached, the tissue is placed on positively charged slides, as per routine prior to H&E staining to highlight the underlying histopathological features, to enable histopathological assessment.

### Step 2: selecting the area of interest

To maximize the number of individual tissue specimens (or areas of interest in a larger tissue—e.g. metastatic tumors) that can fit into one Visium capture array, an experienced pathologist provides a review of a digitally scanned (various manufacturers; 20× or 40× magnification) H&E image and selects the most appropriate whole tissue lesion, or area of interest, according to the specific research question.

### Step 3: FFPE block preparation prior to sectioning

Following Step 1 and 2, on the day of sectioning, blocks are submerged in UltraPure water (ThermoFisher Scientific, 10,977,015) and cooled for at least an hour on a cooling plate set at −4 °C, to ensure the sample is thoroughly rehydrated. This step allows the paraffin and tissue to equilibrate, reducing the risk of tearing, folding or incomplete expansion of the tissue section when floated on the water bath.

### Step 4: tissue slide preparation

An appropriately sized square is marked on the back of a positively charged slide (refer to 10× Genomics user guide #CG000520 for manufacturer recommendations) to guide section placement (NB—other spatial profiling platforms have different size imaging windows. Please refer to respective user guides). The goal here is to have maximal tissue pieces placed in this square that matches the size of the Visium slide 6.5 × 6.5 mm or 11 × 11 mm windows. This allows for all tissue pieces to be transferred to the Visium slide when using the CytAssist instrument (10× Genomics). Since the Visium slide has 2 × windows, this means that 2 × “tissue slides” are prepared.

### Step 5: isolating the area of interest

For each new specimen the microtome blade is repositioned to avoid cross-contamination (NB—specialist microtome training is required before usage). To avoid potential RNA degradation with exposure to the air, the blocks are firstly faced by discarding at least 12 μm (3 × 4 μm) from the surface of the block before a final 4 μm section is cut. During sectioning, the annotated H&E images from Step 2 guides the selection of the area of interest by comparing histopathological features between the section and the annotated reference images. Depending upon your study requirements, this may be whole tumor excisions (including normal tissue) or regions of interest within the tissue section. Excess paraffin is trimmed as much as possible, without damaging the tissue integrity. Regions of interest are dissected from each section by performing dissection directly on the microtome stage using a fresh scalpel blade (Medical and Surgical Requisites, EU-210-1) or using forceps with an angled end (various providers) to perform dissection directly in the water bath.

### Step 6: section expansion on water bath

The isolated tissue section is floated on the water bath at the previously determined suitable temperature (Step 1) for at least 30 s to allow expansion, before being transferred to the marked-up positively charged slide (Step 7). Depending on the sample, the tissue might require a longer time in the water bath to flatten. If the section originates from an aged block, containing too much cutaneous tissue, or was under-fixed, the water bath incubation may be reduced to less than 20 s. Alternatively, the water bath temperature can be adjusted to a lower temperature. Lowering the water bath temperature is a common practice used to prevent the paraffin from over-softening, which reduces water penetration into the tissue that causes section swelling, and limits thermal expansion of the section to preserve tissue integrity.

### Step 7: section transfer

It is essential to clean lab-approved gloves before handling sections for transfer, usually by applying 80% ethanol, rubbing hands thoroughly, and allowing them to air dry to minimize the risk of contamination. When placing tissue sections on the slide, angled forceps are used to guide the tissue placement by gently guiding the section across the water surface. It is also important to clean the forceps with RNase away (or alternatives) and 80% ethanol before section transfer. Contact with the section or slide should be prevented to avoid contamination or section disintegration. This process is repeated until all sections are placed as close as possible within the pre-drawn window(s) (Step 4) (Figure 1a). The marked square drawn on the back of the slide (Step 4) is then removed using 80% ethanol. These curated slides are now ready to enter the standard Visium CytAssist workflow (10× Genomics CG000520, CG000495) (Figures 1b,c).

## Results

**Figure 2:**
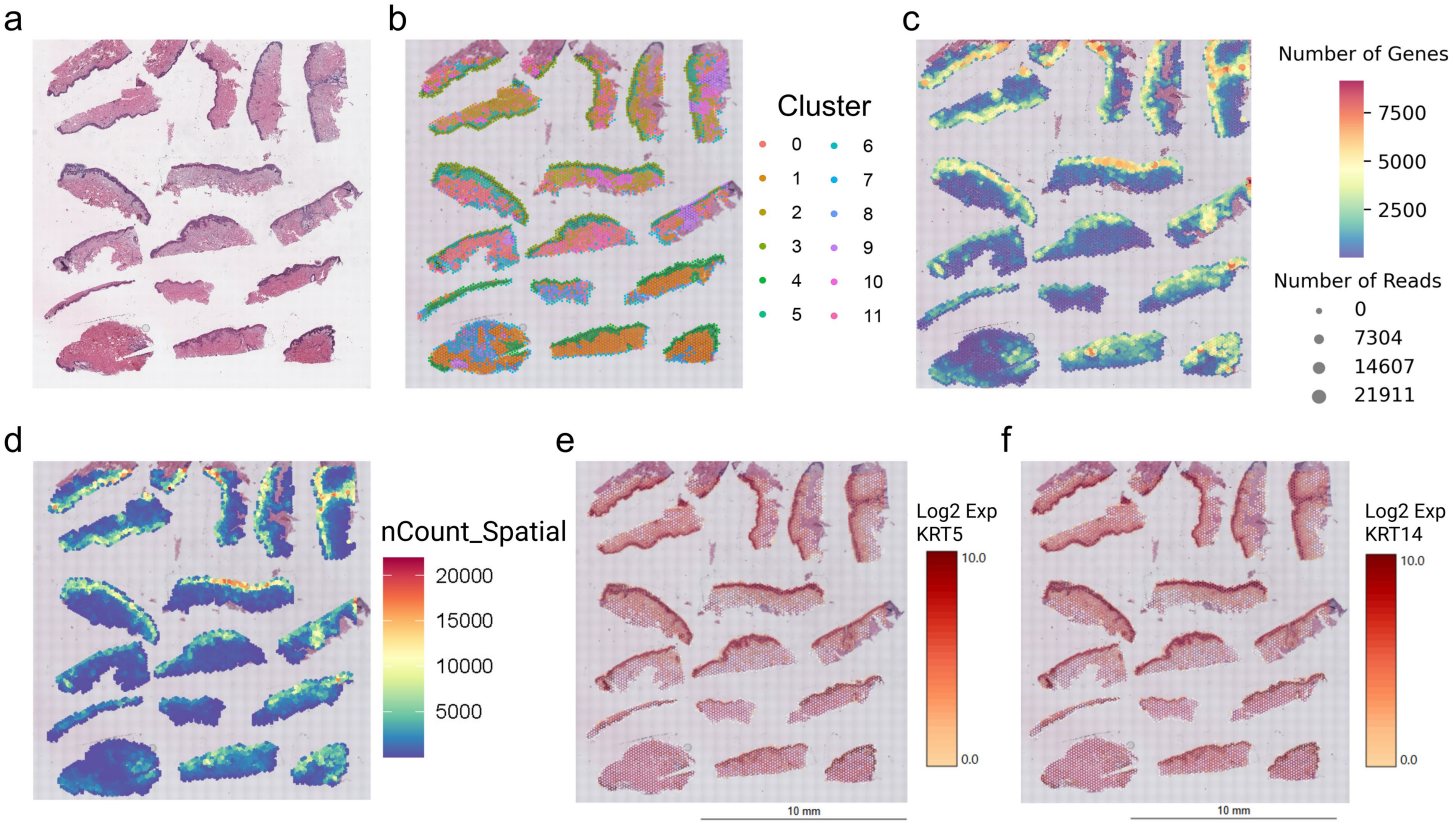
Multiplexed Visium CytAssist capture area and downstream analyses. Multiplexing 17 tissues in one Visium CytAssist capture array reveals the histopathological features of tissue sections through **(a)** H&E images. The sample is subjected to the standard Visium CytAssist workflow. Once the raw data is processed, **(b)** a total of 12 clusters are identified. The quality control metrics demonstrates **(c)** a high number of genes and **(d)** unique molecular identifier in the epidermal regions. Marker genes **(e)** *KRT5* and **(f)** *KRT14* were expressed highly in the epidermal regions. The expression patterns of keratinocyte genes confirm the validity of the clustering analysis.

Using our multiplexing method, we have successfully placed 17 independent tissues in one 11 × 11 mm Visium capture array (Figure 2a). Our tissues were approximately 2 mm in width (bisected from a 4 mm punch biopsies) derived from normal human skin. The total number of tissues that can be placed is dependent upon the study type and sectioning competency. Following the Visium CytAssist workflow as per manufacturers guidelines, each sequencing library was generated to enable the spatial profiling of the FFPE sections.

In our experience, the area covered by tissues must be comparable across all capture arrays to ensure consistent sequencing coverage and uniform sequencing depth across all samples. The required number of sequencing reads depends on the amount of material available within a capture array and the percentage of array area covered by tissues. Sample quantity can be assessed using a Bioanalyzer (Agilent Technologies) as per manufacturers protocol. Array usage can be calculated in QuPath by manually outlining the individual tissue borders and dividing the total multiplexed tissue area by the fiducial capture array area. The required amount of the sequencing pool (800 *fmol* for our sequencing run) and loading volume from each capture array should be confirmed with the sequencing facility prior to the run. In our experiment, 8 multiplexed capture arrays (from 4 × Visium slides) were pooled in one sequencing run. To ensure sufficient coverage, we targeted 25,000 read pairs per spot. The pooled library were then submitted for NovaSeq 6000 (Illumina) sequencing using the S1 100 cycle kit. In the capture area depicted in Figure 1 with 17 independent tissue sections, a total of 5,210 Visium spots were covered, with 18,060 genes detected (mean reads per spot = 30,456; 865 median genes per spot).

In brief, downstream analysis revealed a total of 12 clusters (Figure 2b). The number of genes (Figure 2c) and the total number of unique molecular identifiers per spot (Figure 2d) correlated with the expected gene expression based upon histopathological tissue architecture. As anticipated, there was a higher cell density in the epidermis as compared to the dermis and subcutaneous regions, which corresponded to a higher number of genes per spot. To confirm the validity of clustering, marker genes for keratinocytes (e.g., *KRT5* and *KRT14*) were abundantly expressed in the epidermis, and spatially resolved (Figures 2e,f) in their expected location. The expression patterns of both *KRT5* and *KRT14* correspond to the clustering result, where the keratinocytes were classified within the same cluster. These results validate and demonstrate the successful implementation of multiplexing approach within the Visium CytAssist workflow.

## Discussion

Spatial transcriptomics is one technology that enables molecular profiling of precious pathology specimens. There have been studies demonstrating the primary limitations to scaling up this research are the high costs and the large sample sizes required (7). Our presented method overcomes the limitations and demonstrates the advantages of utilizing a multiplexing approach which allows for processing of larger samples sizes if the same research budget was available (or a significant cost saving if no increase in sample size). Importantly, our approach preserves precious pathology specimens without the need to perform a core biopsy. This approach is applicable to multiple spatial platforms such as GeoMx, CosMx, Visium HD, Xenium *in situ*, PhenoCycler-Fusion, and MERSCOPE (8).

One limitation to multiplexing different tissues is the optimal water bath temperature and section thickness varies from block to block. These differences often arise from the differing fixation times processed in the pathology labs, as well as the diverse tissue types. As a result, achieving consistent conditions can be challenging especially working with older tissue specimens or multiple tissue types, and this variability may introduce additional complications. In particular, possible artifacts arising from this approach lie in the tissue placement on the water bath. Even though the water bath temperature and section thickness are optimal, improper placement in the water bath may cause the section to expand too quickly, fold, or distort due to external factors such as fluctuations in air flow. These effects are more prominent in delicate or aged samples. Therefore, tissue placement guided with the forceps should be handled carefully. Direct contact between the forceps and the tissue section should also be avoided to preserve the section integrity. Additionally, when placing tissues on the slides, paraffin should not be overlapped on the tissue slides to ensure that complete tissue is profiled. These methods are broadly applicable to archival blocks from diverse tissue types and across multiple spatial platforms.

## Data Availability

The original contributions presented in the study are included in the article/supplementary material, further inquiries can be directed to the corresponding author.

## References

[1] Gracia Villacampa E Larsson L Mirzazadeh R Kvastad L Andersson A Mollbrink A . Genome-wide spatial expression profiling in formalin-fixed tissues. Cell Genom. (2021) 1:100065. doi: 10.1016/j.xgen.2021.100065, 36776149 PMC9903805

[2] Stahl PL Salmen F Vickovic S Lundmark A Navarro JF Magnusson J . Visualization and analysis of gene expression in tissue sections by spatial transcriptomics. Science. (2016) 353:78–82. doi: 10.1126/science.aaf2403, 27365449

[3] Zhou R Yang G Zhang Y Wang Y. Spatial transcriptomics in development and disease. Mol Biomed. (2023) 4:32. doi: 10.1186/s43556-023-00144-0, 37806992 PMC10560656

[4] Donovan ML Jhaveri N Ma N Cheikh BB DeRosa J Mihani R . Protocol for high-plex, whole-slide imaging of human formalin-fixed paraffin-embedded tissue using PhenoCycler-fusion. STAR Protoc. (2024) 5:103226. doi: 10.1016/j.xpro.2024.103226, 39031553 PMC11314888

[5] Ganier C Mazin P Herrera-Oropeza G Du-Harpur X Blakeley M Gabriel J . Multiscale spatial mapping of cell populations across anatomical sites in healthy human skin and basal cell carcinoma. Proc Natl Acad Sci USA. (2024) 121:e2313326120. doi: 10.1073/pnas.2313326120, 38165934 PMC10786309

[6] Edqvist PH Fagerberg L Hallstrom BM Danielsson A Edlund K Uhlen M . Expression of human skin-specific genes defined by transcriptomics and antibody-based profiling. J Histochem Cytochem. (2015) 63:129–41. doi: 10.1369/0022155414562646, 25411189 PMC4305515

[7] Srinivasan G Davis MJ LeBoeuf MR Fatemi M Azher ZL Lu Y . Potential to enhance large scale molecular assessments of skin Photoaging through virtual inference of spatial transcriptomics from routine staining. Pac Symp Biocomput. (2024) 29:477–91. doi: 10.1142/9789811286421_0037 38160301 PMC10813837

[8] Oliveira MF Romero JP Chung M Williams SR Gottscho AD Gupta A . High-definition spatial transcriptomic profiling of immune cell populations in colorectal cancer. Nat Genet. (2025) 57:1512–23. doi: 10.1038/s41588-025-02193-3, 40473992 PMC12165841

